# Supplementary material for: Strong yet flexible ceramic aerogel
Source: Nat Commun. 2023 Nov 3;14:7057. doi: 10.1038/s41467-023-42703-7 (PMC10624812; doi:10.1038/s41467-023-42703-7)
Supplement: Supplementary file 1 — Supplementary Information [file 41467_2023_42703_MOESM1_ESM.pdf]

Supplementary Information

Strong yet Flexible Ceramic Aerogel

Lei Su[1], Shuhai Jia[2], Junqiang Ren[3], Xuefeng Lu[3], Sheng-Wu Guo[1], Pengfei Guo[1], Zhixin Cai[1], De Lu[1], Min Niu[1], Lei Zhuang[1], Kang Peng[1], Hongjie Wang[1]**

[1] State Key Laboratory for Mechanical Behavior of Materials, Xi'an Jiaotong University, Xi'an 710049, China.
[2] School of Mechanical Engineering, Xi'an Jiaotong University, Xi'an 710049, China
[3] State Key Laboratory of Advanced Processing and Recycling of Non-ferrous Metal, Department of Materials Science and Engineering, Lanzhou University of Technology, Lanzhou 730050, China
*Corresponding authors. Email: shjia@xjtu.edu.cn; hjwang@xjtu.edu.cn

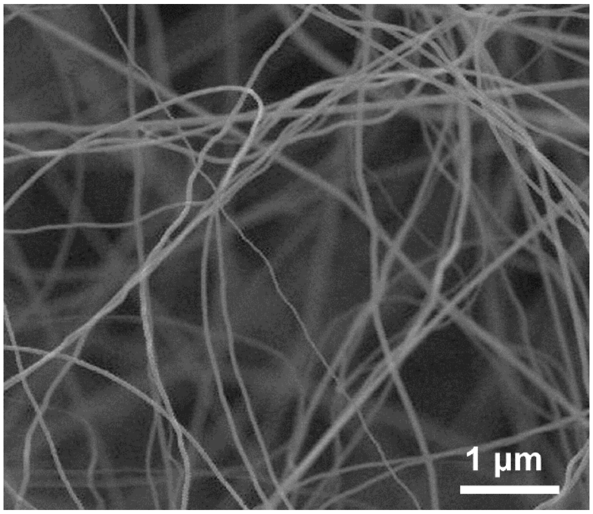

Supplementary Fig. 1 | Microstructure of the raw SiC-SiO_x nanowire aerogel. Random distribution of the nanowire showing the isotropic structure.

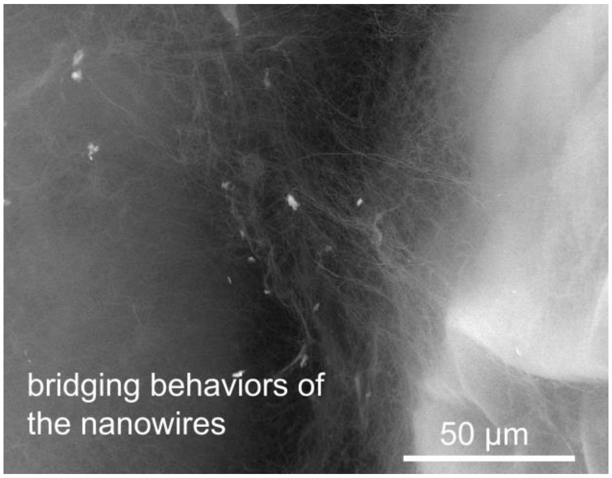

Supplementary Fig. 2 | Microstructure of the interface between neighboring layer during delamination. The bridging behavior of the nanowire at the interface between neighboring layers indicating the good connection.

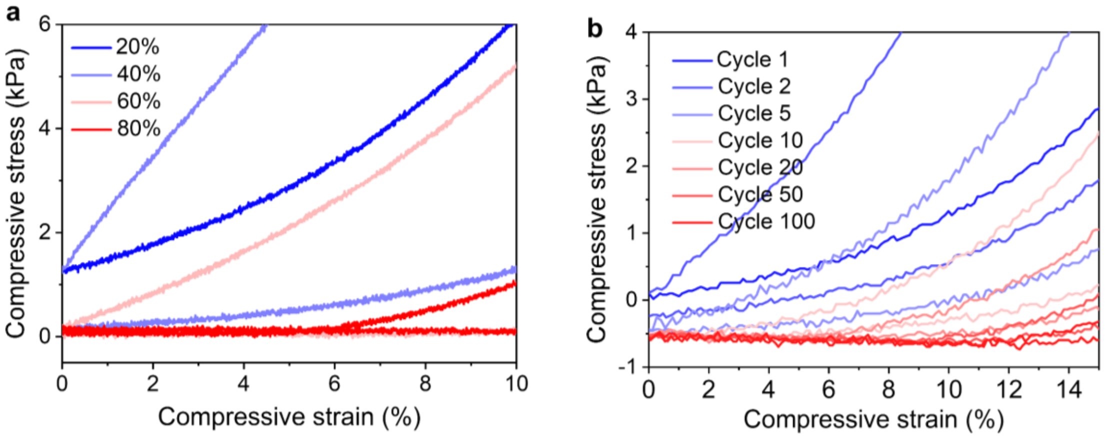

Supplementary Fig. 3 | Compressive tress-strain curves of the laminated aerogel at low strains. a. Consecutive compression at set strains of 20%, 40%, 60% and 80%. **b.** 100 cyclic fatigue compressive tests at set strain of 40%.

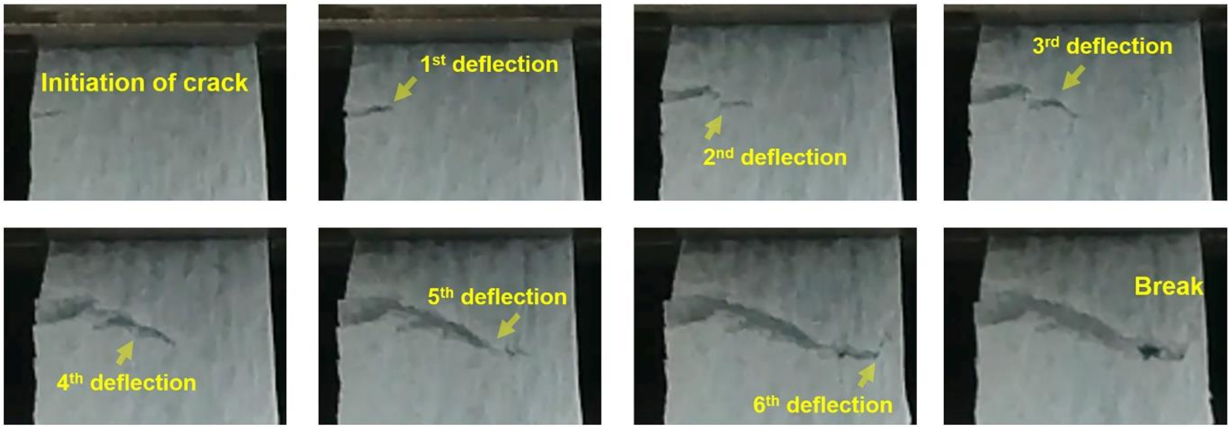

Supplementary Fig. 4 | Initiation and propagation of the crack during tensile deformation. The evolution of the crack morphology showing six times of deflection during the propagation of the crack before break.

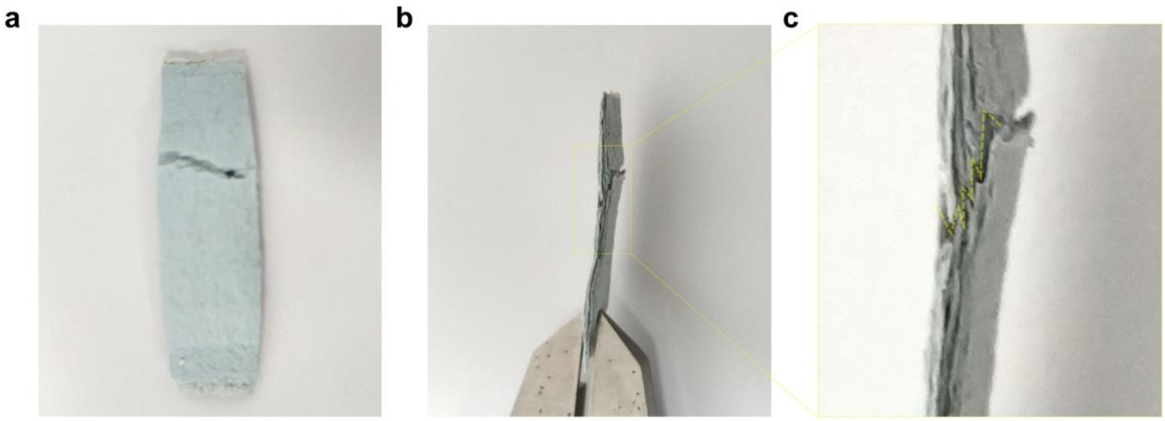

Supplementary Fig. 5 | Macroscopic morphology of the fractured sample after tensile test. a, b. The sample exhibiting its integrity after fracture. **c**. Amplified area in **b**, showing a layer-by-layer fracture behavior.

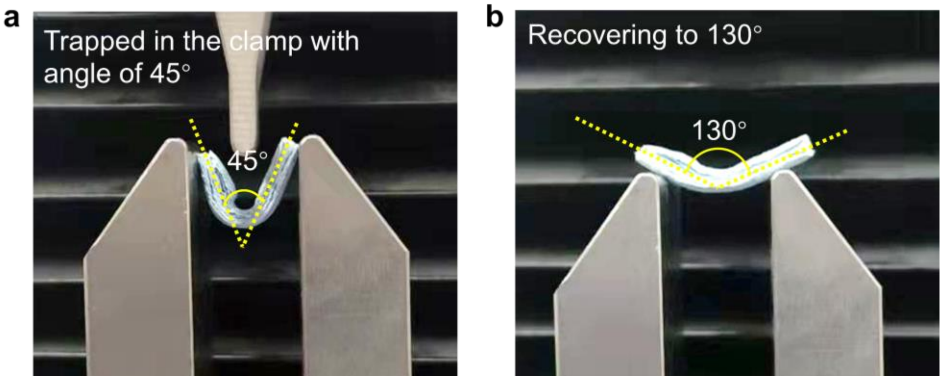

Supplementary Fig. 6 | Recoverable bendability of the laminated aerogel. a. The trapped aerogel sample with a bending angle of 45°. **b.** The trapped sample recovered to 130° after being taken down from the clamp.

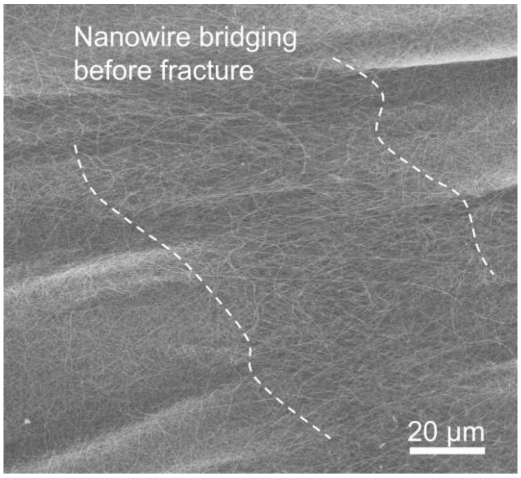

Supplementary Fig. 7 | Formation of nanowire bridge before fracture under tensile deformation. The nanowire bridging behavior indicating a nonbrittle fracture of the laminated aerogel.

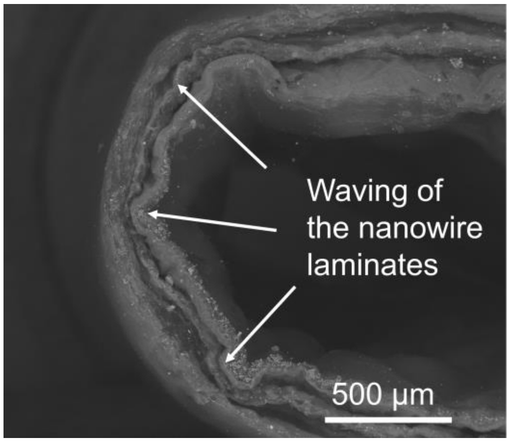

Supplementary Fig. 8 | The microstructure morphology of the present laminated aerogel under bending. The nanowire layers showing obvious waving behavior under bending, indicating its flexibility.

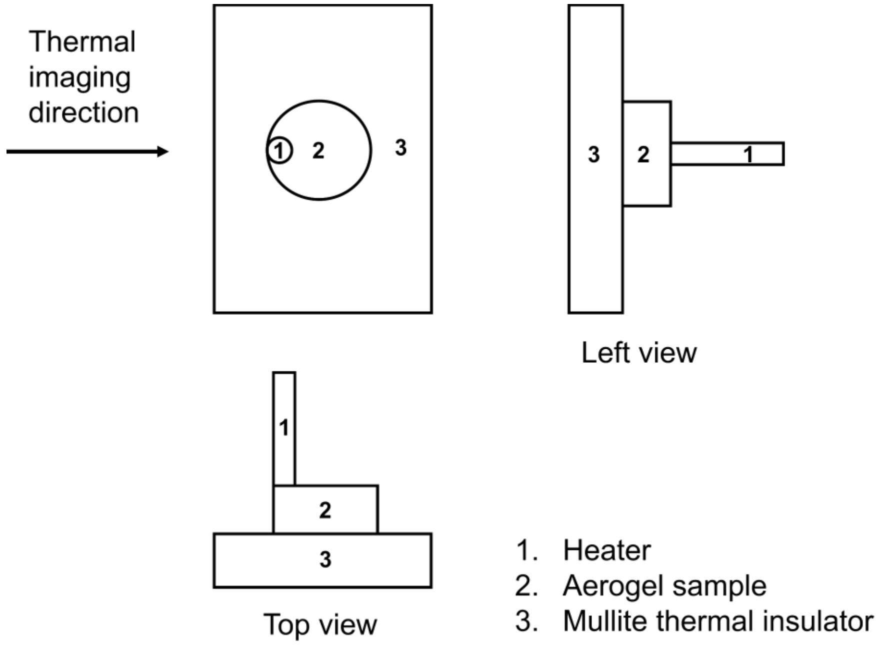

Supplementary Fig. 9 | Illustration of the experimental settings during the observation of the heat transportation behavior in the aerogel sample. A cylinder heater with a diameter of 2 mm was used as the heat source to generate heat on the surface of the aerogel samples. The thermal imager in the front of the heater were used to record the evolution of the 25 °C isothermal lines in the samples during the heating process.

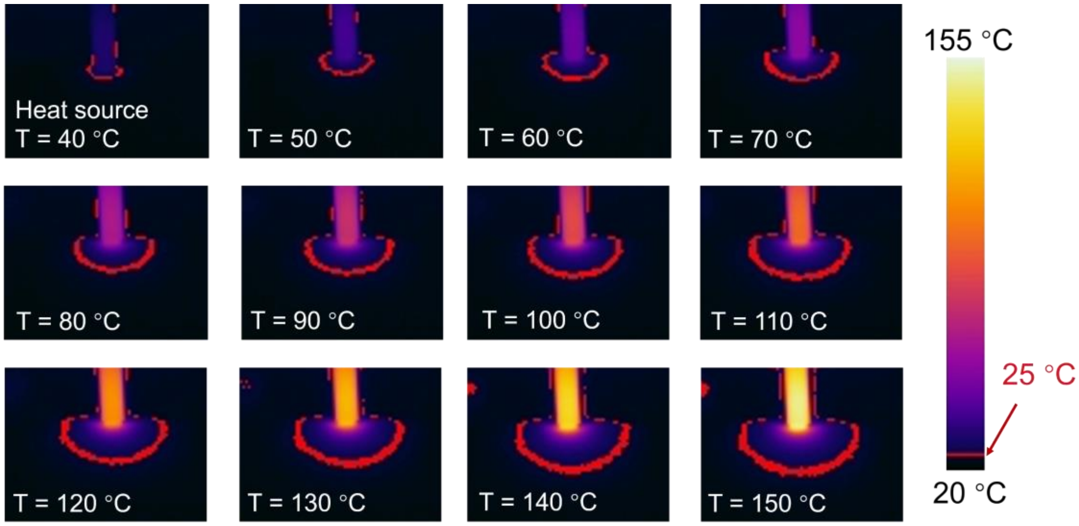

Supplementary Fig. 10 | The evolution of the positions of 25 °C isothermal line in the laminated aerogel with the increase of the temperatures of heat source during the heating process. The morphologies of the isothermal lines obtained by the infrared images indicating an anisotropic thermal conducting behavior.

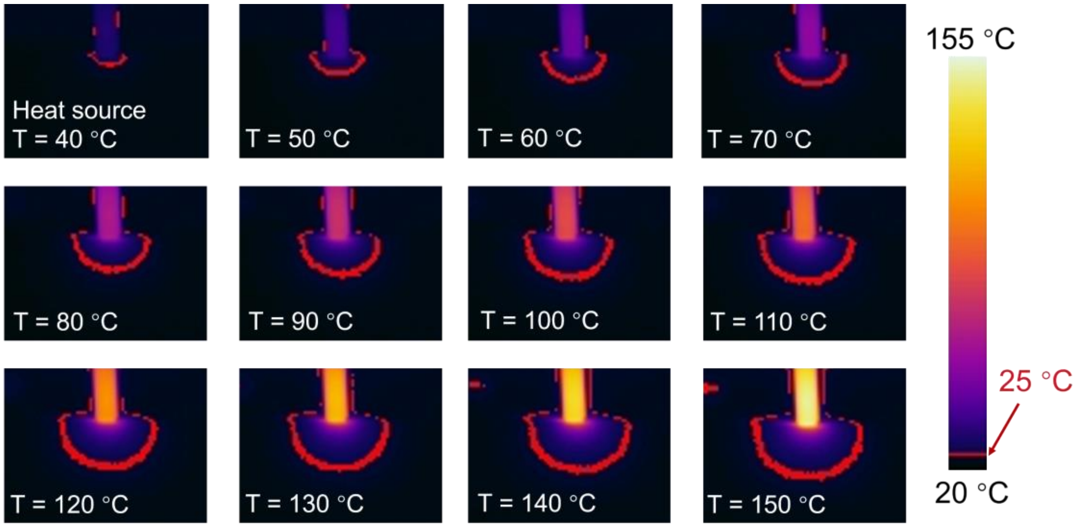

Supplementary Fig. 11 | The evolution of the positions of 25 °C isothermal line in the isotropic aerogel with the increase of the temperatures of heat source during the heating process. The morphologies of the isothermal lines obtained by the infrared images indicating an isotropic thermal conducting behavior.

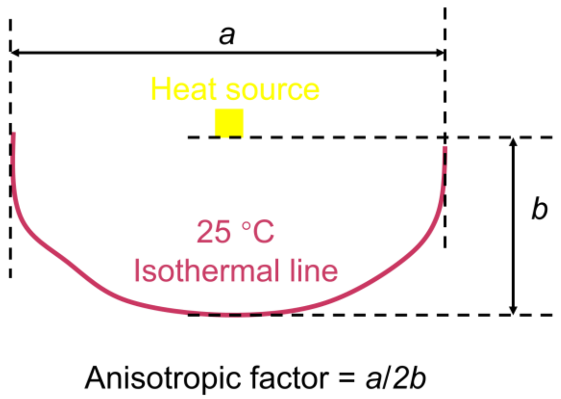

Supplementary Fig. 12 | Illustration of the calculation of anisotropic factor of the shape of 25 °C isothermal lines. Anisotropic factor is equal to the ratio between half of the horizonal length *a* and vertical length *b*.

Supplementary Discussion.

We analyzed the pore size distribution in the laminated aerogel by integrating the N_2 sorption, mercury intrusion porosimetry, and the total pore calculation. N_2 sorption can provide the size information of the mesopores. Mercury intrusion porosimetry can reflect the size information of the micropores. The total pore calculation is a beneficial supplement to the above two methods and can reflect the whole picture of pore size distribution. The details are discussed below.

N_2 sorption measurement. Fig. 2j and k shows the N_2 sorption isotherms at 77 K and pore size distribution of the laminated aerogel derived from Barrett–Joyner–Halenda (BJH) analysis, respectively. The calculated specific surface areas and average pore sizes of the laminated aerogel are 15.4 m^2 g^{-1} and 11.8 nm, respectively. The micropore and mesopore volume per gram, $V_{micro\&meso,}$ is 0.03 cm^3 g^{-1}.

Mercury intrusion porosimetry. In the present case, the laminated aerogel is a highly compressible material. The deformation during the intrusion can influence the results: when the pressure is too high, the structure of the aerogel might be destroyed and some pores could could be compressed or disappear and cannot be detected by mercury intrusion porosimetry. From the compressive property of the laminated aerogel, we can see that the maximum stress can reach an average value of 1255±116.3 kPa at 80% strain. It is sure that before the pressure of mercury reaches more than 1255 kPa the structure of the laminated aerogel is integral, except for the elastic deformation.

Herein, we assume that the pore shape is spherical, then the volume shrinkage ratio, X, during the mercury intrusion process can be calculated by equation 1.

$$X = \frac{\frac{4}{3}\pi R^3 - \frac{4}{3}\pi r^3}{\frac{4}{3}\pi R^3} \qquad (1)$$

Where R is the real pore radius of the pores, r is the tested pore radius. The real pore radius be calculated by equation 2.

$$R = \left(\frac{1}{1\text{-}X}\right)^{\frac{1}{3}} r \qquad (2)$$

Because of the nearly zero Poison ratio, the volume shrinkage at x% compressive strain is about x%. If there is no volume shrinkage, $R=r$. If the volume shrinkage is 60%, then $R=1.35r$, which indicates that the real pore size is 1.35 times that of the tested one. If the volume shrinkage is 80%, then then $R=1.71r$.

Supplementary Fig. 13 shows the relationship between the tested pore size and the applied pressure to the mercury (in the range of 0 to 1255 kPa). It could be seen that the maximum tested pore size is 355 μm (the pressure of mercury is 3.5 kPa) and the minimum tested pore size is 1 μm (the pressure of mercury is 1255 kPa). When the pressure of mercury exceeds 1255, the pore size might be compressed further. And with the further increase of the pressure, the structure might be destroyed. Therefore, the pore sizes measured here are smaller than the real ones and some pores with sizes in the smaller value range might not be tested.

Combining the mercury intrusion porosimetry results displayed in Supplementary Fig. 14, it could be concluded that the pore size in the laminated aerogel is below 355 μm. The macropore volume ratio measured by mercury intrusion porosimetry, $V_{macro\text{-}m}$, is 15.68 cm^3 g^{-1}. Worth noting

that the $V_{macro\text{-}m}$ measured here is smaller than the real one, V_{macro}, due to the volume shrinkage of the aerogel during the test. Therefore, there would be a "volume of missing", $V_{missing}$. And V_{macro} can be written as equation 3.

$$V_{macro} = V_{macro\text{-}m} + V_{missing} \qquad (3)$$

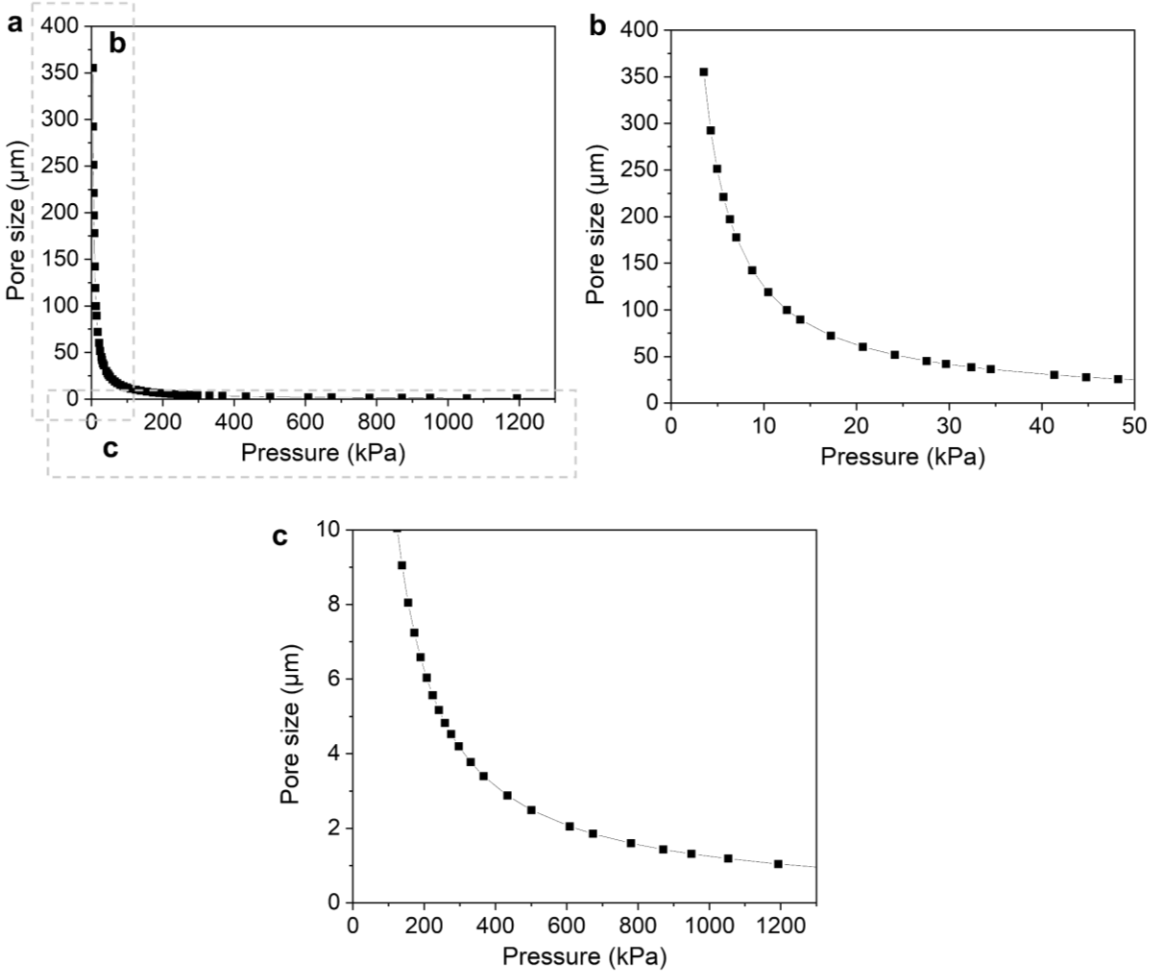

Supplementary Fig. 13 | The relationship between the tested pore size and the applied pressure to the mercury in the range of 0 to 1250 kPa. a. The result showing the range of pore sizes in the laminated aerogel that could be measured by mercury intrusion porosimetry. **b**. Amplified area as marked by "b" in **a**, showing the maximum value of the tested pore size. **c**. Amplified area as marked by "c" in **a**, showing the minimum value of the tested pore size before the fracture of the aerogel during mercury intrusion process.

Total pore calculation and analysis. The bulk density of the present aerogel, ρ_{bulk}, is about 50 mg cm^{-3}. The total pore volume per gram is calculated to be about 20 cm^3 g^{-1} by equation 4.

$$V_{total} = \frac{1}{\rho_{bulk}} \qquad (4)$$

In an aerogel[1],

$$V_{total} = V_{micro\&meso} + V_{macro} + \frac{1}{\rho_{skeletal}} \qquad (5)$$

where $\rho_{skeletal}$ is the density of the skeletal material. The skeletal material in the laminated aerogel is SiC-SiO_x nanowire. The density of SiO_x is 2.8 g cm^{-3}, and the density of SiC is 3.2 g cm^{-3}. Therefore, the density of the skeletal material can be estimated to be 3.0 g cm^{-3}. $\frac{1}{\rho_{skeletal}}$ is calculated to be 0.33 cm^3 g^{-1}.

In the present case,

$$V_{total} = V_{micro\&meso} + V_{macro\text{-}m} + V_{missing} + \frac{1}{\rho_{skeletal}} \qquad (6)$$

then $V_{missing}$ is calculated to be 3.96 cm^3 g^{-1}, and V_{macro} is 19.64 cm^3 g^{-1}.

As a result, in the laminated aerogel, V_{total} is 20 cm^3 g^{-1}, V_{meso} is 0.03 cm^3 g^{-1}, V_{macro} is 19.64 cm^3 g^{-1}. The ratio of mesopore volume in the laminated aerogel is only 0.15%, showing the low mesoporosity in the laminated aerogel. According to Ref. 1, the average pore size of the aerogel calculated by equation 7 is 5.1 μm. These results show that the pores in the laminated aerogel are mainly macropores with sizes below 355 μm and the average pore size of all the pores is about 5.1 μm.

$$D_{average} = \frac{4\times(V_{total} - \frac{1}{\rho_{skeletal}})}{S_{BET}} \qquad (7)$$

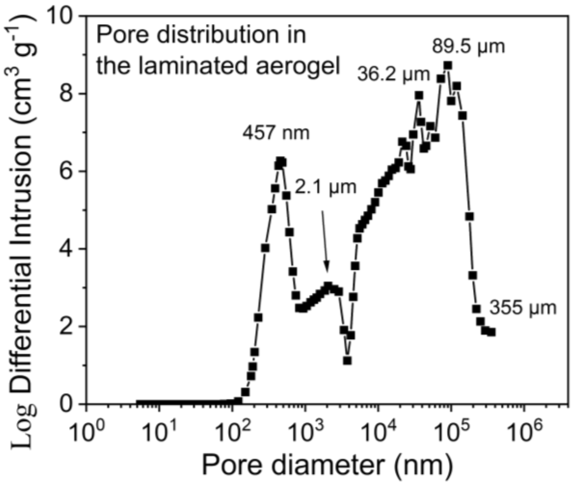

Supplementary Fig. 14 | The tested pore size distribution in the laminated aerogel by mercury intrusion porosimetry. Results showing that the sizes of the pores in the laminated aerogels are below 355 μm.

Supplementary Table 1 | Thermal conductivity of the laminated aerogel. Standard deviation was calculated from five performed measurements.

	**Testing temperature (K)**	**Thermal conductivity (mW m^{-1} K^{-1})**	**Averaged thermal conductivity (mW m^{-1} K^{-1})**	**Standard deviation**
	305.1	39.5	39.3	0.4
	305.0	39.7		
3	305.0	39.2		
4	305.1	38.8		
5	305.1	39.1		

Supplementary Table 2 | Measured relative size of horizonal length *a* and vertical length *b* of the shape of the 25 °C isothermal lines in the laminated aerogel at different heat source temperature during the heating process.

**Temperature of heat source in laminated aerogel (°C)**	***a***	***b***	**Anisotropic factor**
40	0.95	0.3	1.58
50	1.43	0.47	1.52
60	1.75	0.61	1.43
70	1.95	0.68	1.43
80	2.17	0.78	1.39
90	2.34	0.88	1.33
100	2.54	0.95	1.34
110	2.71	1.05	1.29
120	2.8	1.1	1.27
130	2.87	1.16	1.24
140	3.02	1.21	1.25
150	3.24	1.29	1.26

Supplementary Table 3 | Measured relative size of horizonal length *a* and vertical length *b* of the shape of the 25 °C isothermal lines in the isotropic aerogel at different heat source temperature during the heating process.

**Temperature of heat source in isotropic aerogel (°C)**	***a***	***b***	**Anisotropic factor**
40	1.03	0.35	1.47
50	1.48	0.58	1.28
60	1.73	0.76	1.14
70	1.92	0.85	1.13
80	2.06	0.98	1.05
90	2.18	1.11	0.98
100	2.4	1.16	1.03
110	2.55	1.25	1.02
120	2.67	1.36	0.98
130	2.74	1.39	0.99
140	2.86	1.47	0.97
150	3.01	1.54	0.98

Supplementary Table 4 | Average values and standard deviations of the modulus and strength. Standard deviation was calculated from three performed measurements.

	**Sample 1**	**Sample 2**	**Sample 3**	**Average**	**Standard deviation**
**Compressive stress at 80% strain (kPa)**	1263.4	1366.0	1134.0	1255	116.3
**Compressive modulus (kPa)**	201.7	259.5	204.2	222	32.7
**Tensile strength (kPa)**	370.4	492.3	332.8	399	83.4
**Tensile modulus (kPa)**	4979	4801	4775	4855	111.0
**Bending strength (kPa)**	258.5	272.8	250.2	261	11.4

Supplementary references

1. Reichenauer G. Structural characterization of aerogels. *Aerogels handbook*, 449−498 (2011).
