# Supplementary material for: Strong yet flexible ceramic aerogel
Source: Nat Commun. 2023 Nov 3;14:7057. doi: 10.1038/s41467-023-42703-7 (PMC10624812; doi:10.1038/s41467-023-42703-7)
Supplement: Supplementary file 3 — Description of Additional Supplementary Files [file 41467_2023_42703_MOESM3_ESM.pdf]

Description of Additional Supplementary Files:

Supplementary Movie 1: In situ observation of the radial ethanol flow during the evaporation by using an optical microscope.

Supplementary Movie 2: In situ observation of the vertical volume shrinkage and reorientation of the nanowires during evaporation.

Supplementary Movie 3: Macroscopic morphology evolution during the tensile process from 0 to 20% strain showing the ductile deformation behavior.

Supplementary Movie 4: Macroscopic morphology evolution during the buckling and recovery process from 0 to 80% strain.

Supplementary Movie 5: Microstructure evolution of the laminated aerogel during the recovery from 60% strain to the initial state.

Supplementary Movie 6: Reversible bendability of the laminated aerogel in liquid nitrogen.

Supplementary Movie 7: Reversible bendability of the laminated aerogel under butane blow torch.

Supplementary Movie 8: Infrared movie showing the evolution of the positions of 25 °C isothermal line in the laminated aerogel with the increase of the temperatures of heat source during the heating process.

Supplementary Movie 9: Infrared movie showing the evolution of the positions of 25 °C isothermal line in the isotropic aerogel with the increase of the temperatures of heat source during the heating process.
